# Knowledge of and Attitude Toward Disaster Preparedness Among Secondary School Students in the Western Region of Saudi Arabia

**DOI:** 10.7759/cureus.33926

**Published:** 2023-01-18

**Authors:** Safa H Alkalash, Ethar H Alhashmi Alamer, Adhwaa M Allihyani, Atheer S Alhazmi, Razan M Alharthi, Amani M Bugis

**Affiliations:** 1 Community and Family Medicine, Umm Al-Qura University, Al-Qundufah, SAU; 2 Family Medicine, Menoufia University, Shebin Alkom, EGY; 3 College of Medicine, Umm Al-Qura University, Makkah, SAU

**Keywords:** secondary school students, saudi arabia, preparedness, knowledge, disaster management, attitude

## Abstract

Background

Disaster is any unexpected event that leads to significant harm exceeding the capacity of the affected community for coping. Unfortunately, disasters have increased during the last few years globally. Knowledge and attitude of people are the main steps for the success of disaster preparedness and management. Thus, this study was conducted to assess the level of knowledge and attitude about disaster preparedness among secondary school students in the western region of Kingdom of Saudi Arabia.

Methodology

A cross-sectional, survey-based study was conducted on a sample of 726 secondary school students who were chosen from the western region of Saudi Arabia through a multistage sampling approach. The data were collected using a validated, self-administered, electronic questionnaire comprising 34 items. All data were analyzed using SPSS version 23 (IBM Corp., Armonk, NY, USA).

Results

A total of 726 respondents were recruited in this study. The majority of the respondents were females (79.5%), and about half (53.9%) belonged to the 17-18-year age group. About half of the participants had a good understanding of disaster preparedness. The most common source of their information was social media (78.8%). Despite the majority of the study population showing a positive attitude toward disaster preparedness, only 34.2% of the respondents were previously involved in a disaster drill(s) or workshop(s). Students’ knowledge of disaster preparedness was significantly associated with their gender (p < 0.001), father’s and mother’s education (p < 0.001 for each), father’s occupation (p = 0.005), and average monthly income (p < 0.001). The average monthly income of parents was shown to be significantly correlated with attitude scores toward disaster management preparedness.

Conclusions

This study revealed that the perceived knowledge regarding disaster preparedness among secondary school students in the western region of Saudi Arabia was fair with a high level of positive attitude toward it. Educated parents, employed fathers, and high family income were associated with good knowledge of students. Disaster response training simulation for students is highly recommended to be involved in the curricula.

## Introduction

The International Federation of Red Cross Society (IFRCS) defines disasters as any unforeseen event leading to dysfunction in society resulting in economic, property, and human losses to an extreme limit where the society can no longer handle the adverse effects [1]. The extent, frequency, and type of hazards differ by geography, ethnicity, and economic capacity [2]. Floods, earthquakes, heat waves, and droughts are some examples of natural disasters [3]. However, occupational hazards, infectious disease outbreaks, terrorist attacks, and others are manmade disasters [2].

Disasters cause extremely damaging effects. First, they increase the mortality rate as they cause massive injuries such as blunt trauma and crush-related injuries. In 2017, the worldwide disaster database recorded 335 natural disasters worldwide, affecting over 95.6 million people. Of those, 9,697 died, 58% of whom were from Asia, costing approximately US $335 billion of material damage. Second, they destroy property and cause disruption to economic and social infrastructure. Finally, they have a negative impact on the physical, mental, and social well-being of humans [4].

Despite the fact that the Kingdom of Saudi Arabia (KSA) is geologically stable, a variety of severe climate-related conditions, such as floods, heatwaves, and droughts, are possible. Moreover, the abundance of natural disasters in the KSA can be likely due to the geographic diversity as the country has some mountainous or valley areas in addition to coastal regions while other regions are extremely dry deserts [5].

Most flood hazards in the KSA are caused by a combination of natural conditions (heavy rainfall and climate change) and human interference (poor drainage systems and unplanned urban expansion). Nowadays, heavy rainfall events attack different areas of the KSA (Jeddah City in 2009 till today). The severely hit areas were generally in the western part of the country, particularly in the city of Jeddah between November 2009 and January 2011 [6,7].

Disaster preparedness is defined by the United Nations International Strategy for Disaster Reduction (UNISDR) as the knowledge, capabilities, and actions of governments, organizations, community groups, and individuals to effectively anticipate, respond to, and recover from, the impacts of likely, imminent, or current hazard events or conditions [8]. Therefore, disaster prevention and preparedness such as risk assessment and multidisciplinary management strategies at all system levels are critical because all these measures and strategies will help national governments manage the risks of continuing threats from natural and deliberate hazards.

The education sector is one of the most important sectors for disaster preparedness where disasters affect a large scale of the population such as students, teachers, and other school components [4]. The main cause for this is the presence of more than six million children younger than 18 years living in KSA. These children spend a long time on their school premises, and the average school-aged child spends six to eight hours of his or her day in school [9]. Therefore, it is crucial to prepare the school community and students by developing educational plans and instructions while keeping important considerations in mind such as overcrowding, event access points (entry and exit), fire safety instructions, weather hazards, and emergency response.

As disasters strike without warning, the entire school community including students needs to be familiar with disaster management. However, disaster preparedness is considered one of the key steps to reaching the safe side without missing many victims.

knowledge of the school community (those working, teaching, and learning in a school, i.e., the administrators, faculty, staff, and students) is of the utmost importance because by recognizing environmental conditions they can be more alert about and predict what will happen to the environment and translate this knowledge into acceptable attitudes to manage it. Furthermore, attitudes about natural disasters are very critical for the school community because both knowledge and attitude can direct their behavior to deal with such conditions without being anxious or panicking.

In 2015, a survey was conducted to determine and characterize the elements that affect the knowledge and perception of secondary school students in Belgrade about earthquakes as natural disasters. The findings demonstrated how secondary school students’ perspectives are influenced by the informational sources used to describe natural disasters and their potentially dangerous effects [10].

At the end of the year 2022 and the beginning of 2023, Makkah and Al-Madinah regions were exposed to heavy rainfall that led to vigorous floods which destroyed considerable property and houses and accounted for a number of traffic accidents with many victims. These floods forced policymakers to shift the educational process from school attendance to virtual education to save students’ lives. Little is known about disaster preparedness among its population including school students. Therefore, this study was conducted to explore the knowledge of and attitude toward disaster preparedness among secondary school students in the western region of Saudi Arabia.

## Materials and methods

Study design

This cross-sectional study was conducted to determine the knowledge of and attitude toward disaster preparedness among secondary school students in the western region of Saudi Arabia over a five-month period from September 2022 to January 2023. A pretested validated questionnaire was used to collect the research data.

Sample size

‏The sample size was estimated using Epi Info™ (Centers for Disease Control and Prevention, Atlanta, GA) based on the total number of secondary school students in the western region of Saudi Arabia (163,609) and the percentage of good knowledge about disaster preparedness (61.8%) at 95% confidence interval (CI) and a 5% margin of error [11]. Thus, the minimum required sample size was calculated to be 362 participants.

Study setting

The study was conducted in secondary schools in the western region of Saudi Arabia, including Makkah, Jeddah, Al-Madinah, Al-Qunfudah, Taif, and Al-Ardiyat. According to a report from the General Authority for Statistics, KSA [12], there are 1,530 secondary schools in the western region distributed in the Makkah (1,148) and Al-Madinah regions (382).

This study used a multistage sampling approach to select the study sample. Stage one was to select six governorates from the western region (Makkah, Jeddah, Al-Madinah, Al-Qunfudah, Taif, and Al-Ardiyat) from which the schools were chosen. Stage two was to select 14 secondary schools from the previously mentioned governorates: 10 secondary schools from the Makkah region and four secondary schools from the Al-Madinah region.

Procedure and tool for data collection

Data were collected from a cluster sample of 726 secondary school students from the selected schools through an online questionnaire created as a Google Form. The link to this survey was distributed to respondents via WhatsApp and Telegram groups of students in the schools. Furthermore, they were asked to share the survey link with other schoolmates to increase the response rate through a snowball sampling technique.

The questionnaire was designed by the study researchers using a three-step procedure, namely, literature review, content generation, and focus group discussion, followed by pre-testing. In the first step, the researchers gathered information related to disaster preparedness by conducting an in-depth literature review. Next, the relevant information was abstracted, and generated items that were relevant to the research question were drafted in the Arabic language as a 34-item questionnaire. The items were organized within the questionnaire with the help of an expert panel consisting of three members, followed by pre-testing through a pilot study.

Pilot study

A total of 40 responses were analyzed to check the language clarity and understandability of the questions. All data from this pilot study were used only to direct the investigators on whether this survey would be valid or, and were not included in the main study results. Finally, the test-retest technique was used to ensure the reliability of the designed survey.

The finalized draft of the used questionnaire was categorized into three sections. The first section consisted of 11 items to assess the socioeconomic data of the respondents such as age, gender, residence, academic level, fathers’ and mothers’ education and occupation, family size, and income. The second section involved 17 questions to evaluate their knowledge about disasters such as whether they had heard about disasters; their definition, causes, and forms; and the personnel responsible for their management. The third section comprised six items to assess their attitude toward disaster preparedness such as the importance of conducting training for students about measures to manage different forms of disasters and the availability of emergency plans within each school.

A common scoring method was used for knowledge questions (17) as follows: 1 point was given for correct answers, and 0 for incorrect and neutral answers. A participant who scored less than 50% correct answers was considered as having poor knowledge, 50-80% considered as fair knowledge, and those who scored at least 80% were considered as having good knowledge. Attitude scoring was as follows: 2 points for agree, 1 point for neutral, and 0 points for disagree. A participant who scored 80% or more was considered as having a positive attitude, while those below this percentage were considered as having a negative attitude.

Ethical considerations

Ethical approval was provided by the Medical Research and Ethical Committee of the College of Medicine of Umm Al-Qura University, Makkah (reference number: HAPO-02-K-012-2022-11-1235). The confidentiality of the anonymously collected data was maintained.

Data analysis

The data collected were entered in Microsoft Excel and analyzed statistically using SPSS version 23 (IBM Corp., Armonk, NY, USA). All questionnaire items needed to be completed by the respondents to submit their responses; therefore, there were no missing data. Qualitative data were expressed as numbers and percentages, and quantitative data were presented as means and standard deviations. The chi-square test and Fisher’s exact test were used to examine the association between the two groups. A p-value of less than 0.05 was considered significant.

## Results

In this study, a total of 726 respondents were included, and the majority of them were females (79.5%). More than half of the participants were aged between 17 and 18 years (53.9%). The vast majority of the participants were Saudis (89.8%). According to the geographic distribution of respondents, most (89.8%) were from the Makkah region and its provinces. Moreover, the highest percentage of respondents were in the second-grade academic year (42.6%), followed by the third-grade year (36.6%). Concerning fathers’ and mothers’ education, most had achieved a university degree or above. Regarding occupation, more than half of the fathers were employed (57.3%), while most mothers were housewives (58.7%). Regarding the number of family members, most participants had six or more family members (61.6%). Furthermore, the highest percentage of respondents had 10,000-20,000 as the average monthly income (SAR) of their parents (Table 1).

**Table 1:** Sociodemographic characteristics of the secondary school students (n = 726). All data are expressed in numbers and percentages. SAR = Saudi Arabia riyal

Variable	Categories	Frequency	Percentage
Gender	Male	149	20.5
	Female	577	79.5
Age (in years)	<15	29	4.0
	15–16	258	35.5
	17–18	391	53.9
	19–20	31	4.3
	>20	17	2.3
Nationality	Saudi	652	89.8
	Non-Saudi	74	10.2
Region	Makkah region and its provinces	643	88.6
	Al-Madinah region and its provinces	83	11.4
Academic year	First grade	151	20.8
	Second grade	309	42.6
	Third grade	266	36.6
Father’s education	Illiterate	17	2.3
	Read	60	8.3
	Secondary or less	260	35.8
	Diploma	57	7.9
	University/above	332	45.7
Mother’s education	Illiterate	41	5.6
	Read	97	13.4
	Secondary or less	208	28.7
	Diploma	79	10.9
	University or above	301	41.5
Father’s occupation	Unemployed	37	5.1
	Employee	416	57.3
	Business	94	12.9
	Retired	179	24.7
Mother’s occupation	Housewife	426	58.7
	Employee	213	29.3
	Business	26	3.6
	Retired	61	8.4
Number of family members	Less than 3	32	4.4
	3–5	247	34.0
	6 and more	447	61.6
Average monthly income (SAR) of their parents	<5,000	156	21.5
	5,000–9,000	163	22.5
	10,000–20,000	282	38.8
	>20,000	125	17.2

About half of the participants had a good level of knowledge (53.9%). Additionally, the majority of respondents had heard about disasters (80.3%) but less than half were taught about disaster planning (46.0%). Most participants (87.6%) perceived disasters as an imbalance between the demands caused by events and available resources. Most respondents thought that one day their city and school might be affected by disasters (69.0% and 71.8%, respectively). In addition, the vast majority of them (92.1%) recognized disaster planning as preparedness for what might be needed to be done, and how it will be done, both before and after disasters. About 77.1% of the participants agreed that the surrounding hazards that most likely cause disasters in their school/city must be identified and dealt with. Furthermore, most respondents (85.5%) believed that both health and non-health professional employees in the city should be included in disaster management. The majority of the study population (89.4%) recognized that disaster management is an integral collaborative action of different agencies such as hospitals, local health authorities, civil defense, government, and others, as demonstrated in Table 2.

**Table 2:** Knowledge about disaster preparedness. All data are expressed in numbers and percentages.

Items	Yes		No	
	N	%	N	%
Previously heard about the disaster concept	583	80.3	143	19.7
Knew about disaster planning	334	46.0	392	54.0
Disaster is an imbalance between the demands caused by events and available resources	636	87.6	90	12.4
Disasters can be either natural or man-made	676	93.1	50	6.9
One day his/her city might be affected by a disaster	501	69.0	225	31.0
One day his/her school might be affected by a disaster	521	71.8	205	28.2
Disaster planning is to prepare regarding what might need to be done and how it should be done, both before and after disasters	669	92.1	57	7.9
The surrounding hazards that most likely cause disaster to his/her school/city must be identified and dealt with	560	77.1	166	22.9
Knew about the immediate evacuation process during disasters in the school	228	31.4	498	68.6
Knew about special doors for exit during an evacuation process	210	28.9	516	71.1
Knew what they should do when there is an earthquake	430	59.2	296	40.8
Knew what is the safest area to go to during floods	326	44.9	400	55.1
Disaster management includes both health and non-health professional employees in the city	621	85.5	105	14.5
Disaster management is an integral collaborative action of different agencies such as hospitals, local health authorities, civil defense, government, and others	649	89.4	77	10.6

**Figure 1:**
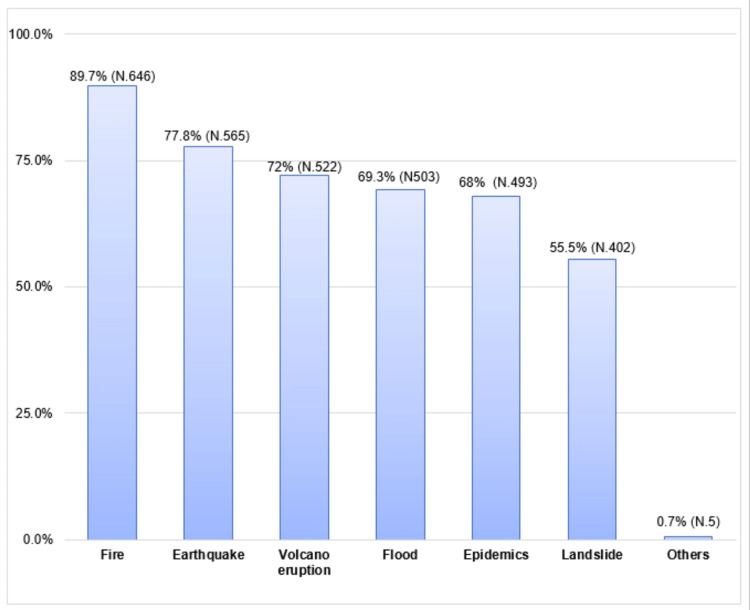
The types of disasters.

Fires were the most commonly recognized type of disaster by the respondents (N = 646, 89.7%), followed by earthquakes (N = 565, 77.8%), volcano eruptions (N = 522, 72.0%), floods (N = 503, 69.3%), epidemics (N = 493, 68 %), and landslides (N = 402, 55.5%) (Figure 1).

**Figure 2:**
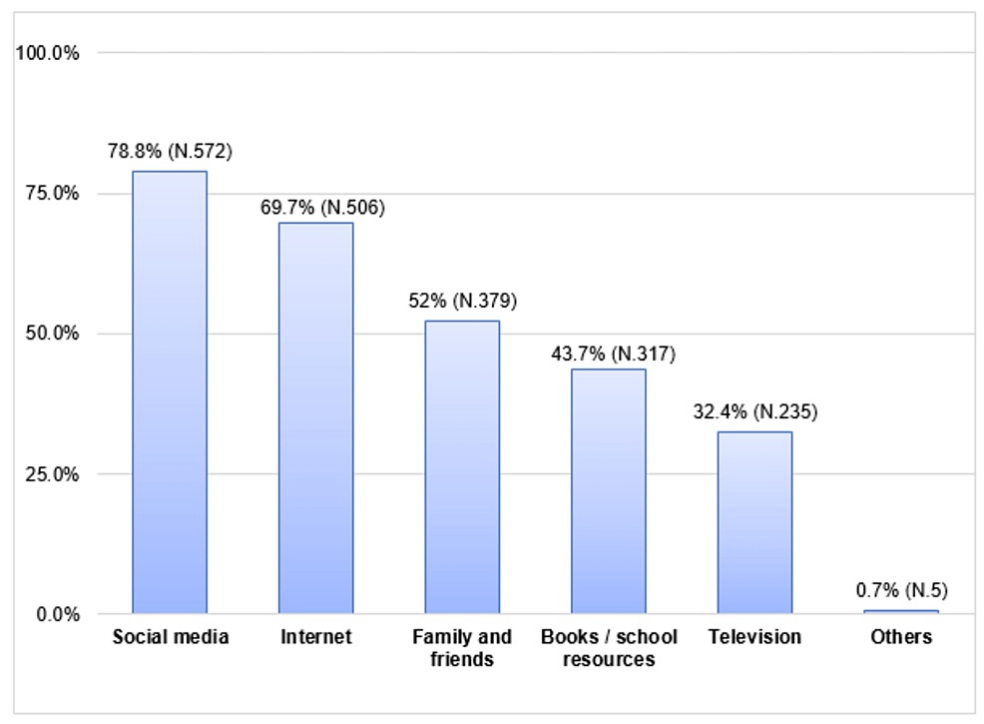
Source of information about disaster preparedness.

Social media was the most frequent source of information (N = 572, 78.8%), followed by the internet (N = 506, 69.7%), family and friends (N = 379, 52.3%), books and school resources (N = 317, 43.7%), and television (N = 235, 32.4%), as shown in Figure 2.

The majority of the study population showed a positive attitude toward disaster preparedness (95.9%), whereas only 4.1% showed a negative attitude. Most respondents (87.1%) agreed that training in disaster planning should be done in their school/city, and most of them (89.9%) believed that active training is necessary. Moreover, most participants agreed on the importance to have a disaster management committee in their school/city (88.2%) and to know their duties and roles during disaster response in their school (92.7%). Furthermore, more than 90% of respondents agreed that training through stimulation exercises, drills, or workshops should be provided to improve disaster management (Table 3).

**Table 3:** Attitude toward disaster preparedness. All data are expressed in numbers and percentages.

Statement	Agree		Neutral		Disagree	
	n	%	n	%	n	%
Training in disaster planning should be taught in the school/city	632	87.1	71	9.8	23	3.2
Training in disaster planning is necessary	653	89.9	48	6.6	25	3.4
It is necessary to have an emergency plan in any school/city for any anticipated hazards	675	93.0	34	4.7	17	2.3
It is necessary to have a disaster management committee in any school/city	640	88.2	67	9.2	19	2.6
Students should know their duties and roles during disaster response in their school	673	92.7	33	4.5	20	2.8
To improve disaster management training through stimulation exercises, drills, or workshops should be provided	657	90.5	49	6.7	20	2.8

**Figure 3:**
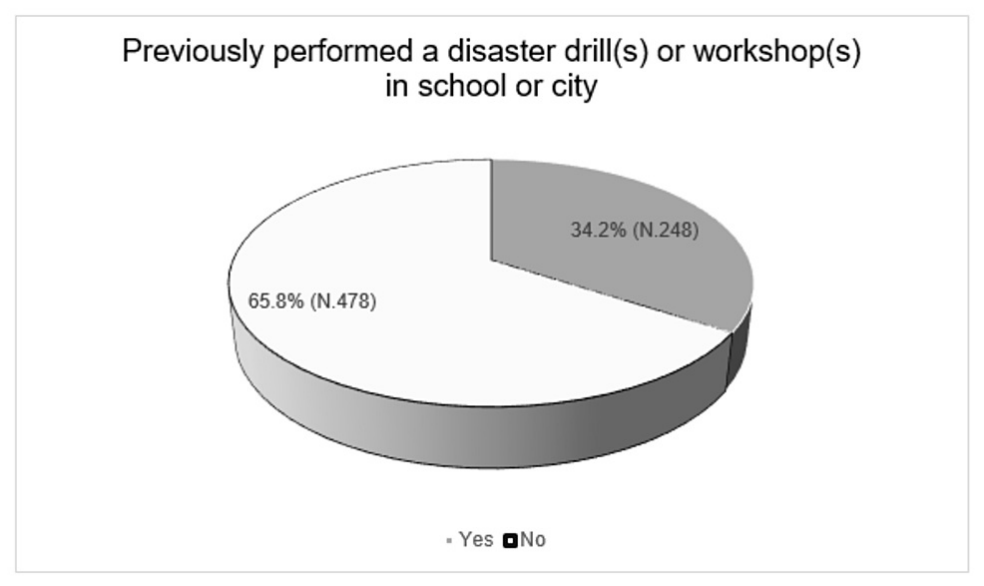
Previously performed a disaster drill(s) or workshop(s) in the school or city.

Only 248 respondents (34.2%) were already involved in a disaster drill(s) or workshop(s) in the school or city (Figure 3).

There were significant relationships between knowledge about disaster management preparedness and gender (p < 0.001), fathers’ education (p < 0.001), mothers’ education (p < 0.001), fathers’ occupation (p = 0.005), and average monthly income (p < 0.001). Females were significantly associated with a higher level of knowledge than males. Participants whose fathers and mothers were illiterate showed the lowest level of knowledge about disaster management preparedness. Moreover, respondents with employed fathers showed better knowledge than others. Moreover, participants who had a 10,000-20,000 average parental monthly income were significantly associated with a better level of knowledge.

The current results showed that only the average monthly income was significantly associated with the attitude about disaster management preparedness. Respondents who had a 10,000-20,000 parental average monthly income were significantly associated with a more positive attitude toward disaster management preparedness (p = 0.040) (Table 4).

**Table 4:** Variables associated with knowledge and attitude about disaster preparedness. *: P-value <0.05 is statistically significant. All data are expressed in numbers and percentages. SAR = Saudi Arabia riyal

Variable	Frequency of good/fair and poor knowledge			P-value	Frequency of positive and negative attitude		P-value
	Poor (n = 18)	Fair (n = 317)	Good (n = 391)		Negative (n = 30)	Positive (n = 696)	
	n (%)				n (%)		
Gender							
Male	10 (6.7)	81 (54.4)	58 (38.9)	<0.001*	10 (6.7)	139 (93.3)	0.076
Female	8 (1.4)	236 (40.9)	333 (57.7)		20 (3.5)	557 (96.5)	
Age (in years)							
<15	0 (0.0)	14 (48.3)	15 (51.7)	0.378	3 (10.3)	26 (89.7)	0.285
15–16	2 (0.8)	112 (43.4)	144 (55.8)		7 (2.7)	251 (97.3)	
17–18	13 (3.3)	171 (43.7)	207 (52.9)		18 (4.6)	373 (95.4)	
19–20	2 (6.5)	14 (45.2)	15 (48.4)		1 (3.2)	30 (96.8)	
>20	1 (5.9)	6 (35.3)	10 (58.8)		1 (5.9)	16 (94.1)	
Nationality							
Saudi	16 (2.5)	285 (43.7)	351 (53.8)	0.990	29 (4.4)	623 (95.6)	0.244
Non-Saudi	2 (2.7)	32 (43.2)	40 (54.1)		1 (1.4)	73 (98.6)	
Region							
Makkah	16 (2.5)	282 (43.9)	345 (53.7)	0.955	30 (4.7)	613 (95.3)	0.069
Al-Madinah	2 (2.4)	35 (42.2)	46 (55.4)		0 (0.0)	83 (100.0)	
Academic year							
First grade	3 (2.0)	63 (41.7)	85 (56.3)	0.513	4 (2.6)	147 (97.4)	0.418
Second grade	5 (1.6)	138 (44.7)	166 (53.7)		12 (3.9)	297 (96.1)	
Third grade	10 (3.8)	116 (43.6)	140 (52.6)		14 (5.3)	252 (94.7)	
Father’s education							
Illiterate	2 (11.8)	12 (70.6)	3 (17.6)	<0.001*	3 (17.6)	14 (82.4)	0.056
Read	4 (6.7)	33 (55.0)	23 (38.3)		3 (5.0)	57 (95.0)	
Secondary or less	7 (2.7)	123 (47.3)	130 (50.0)		12 (4.6)	248 (95.4)	
Diploma	0 (0.0)	21 (36.8)	36 (63.2)		1 (1.8)	56 (98.2)	
University/above	5 (1.5)	128 (38.6)	199 (59.9)		11 (3.3)	321 (96.7)	
Mother’s education							
Illiterate	3 (7.3)	27 (65.9)	11 (26.8)	<0.001*	2 (4.9)	39 (95.1)	0.357
Read	6 (6.2)	47 (48.5)	44 (45.4)		7 (7.2)	90 (92.8)	
Secondary or less	4 (1.9)	105 (50.5)	99 (47.6)		7 (3.4)	201 (96.6)	
Diploma	1 (1.3)	24 (30.4)	54 (68.4)		1 (1.3)	78 (98.7)	
University or above	4 (1.3)	114 (37.9)	183 (60.8)		13 (4.3)	288 (95.7)	
Father’s occupation							
Unemployed	2 (5.4)	23 (62.2)	12 (32.4)	0.005*	4 (10.8)	33 (89.2)	0.125
Employee	6 (1.4)	161 (38.7)	249 (59.9)		13 (3.1)	403 (96.9)	
Business	3 (3.2)	44 (46.8)	47 (50.0)		4 (4.3)	90 (95.7)	
Retired	7 (3.9)	89 (49.7)	83 (46.4)		9 (5.0)	170 (95.0)	
Mother’s occupation							
Housewife	13 (3.1)	198 (46.5)	215 (50.5)	0.242	17 (4.0)	409 (96.0)	0.215
Employee	3 (1.4)	80 (37.6)	130 (61.0)		6 (2.8)	207 (97.2)	
Business	0 (0.0)	12 (46.2)	14 (53.8)		2 (7.7)	24 (92.3)	
Retired	2 (3.3)	27 (44.3)	32 (52.5)		5 (8.2)	56 (91.8)	
Number of family members							
Less than 3	0 (0.0)	21 (65.6)	11 (34.4)	0.068	0 (0.0)	32 (100.0)	0.389
3–5	4 (1.6)	110 (44.5)	133 (53.8)		9 (3.6)	238 (96.4)	
6 and more	14 (3.1)	186 (41.6)	247 (55.3)		21 (4.7)	426 (95.3)	
Average monthly income (SAR) of their parents							
<5,000	8 (5.1)	78 (50.0)	70 (44.9)	<0.001*	11 (7.1)	145 (92.9)	0.040*
5,000–9,000	4 (2.5)	89 (54.6)	70 (42.9)		5 (3.1)	158 (96.9)	
10,000–20,000	3 (1.1)	105 (37.2)	174 (61.7)		6 (2.1)	276 (97.9)	
>20,000	3 (2.4)	45 (36.0)	77 (61.6)		8 (6.4)	117 (93.6)	

## Discussion

This study elucidated the level of knowledge and attitude about disaster management preparedness among secondary school students in the western region of KSA, as knowledge of disasters is an important component in preparing students to face them, particularly natural disasters. Hence, this knowledge should be provided as early as feasible [13].

This study revealed that half of the respondents had a good level of knowledge about disaster management. This finding is slightly lower than that observed in an Indonesian study which reported that the degree of preparedness of class XII students was 74% in the ready category [14]. This discrepancy between both findings is likely related to the difference between both study settings as the Indonesian study was performed among high school students in Indonesia where natural disasters are a common issue compared to KSA. Therefore, knowledge about this topic among Indonesian students was higher.

However, an earlier study in KSA found that respondents had a weak-to-moderate understanding of disaster preparedness [15]. Furthermore, according to another survey in Kashmir valley, there is a widespread lack of information among students on disaster awareness and preparedness [16]. The reason for the higher percentage of good knowledge among the current study respondents in comparison to the previous two studies may be the availability of modern sources of information in the form of social media and more internet coverage.

In Yemen, a recent study reported that the levels of knowledge, positive attitude, and readiness to practice disaster management and preparedness among healthcare students were moderate [17].

On analyzing the findings from the above-mentioned studies, there is an urgent need to promote awareness levels among students to make them more familiar with disaster preparedness plans and to establish disaster management programs as mandatory in all schools.

Furthermore, although the majority of respondents (80.3%) in this study had heard of disasters, fewer than half (46%) had been taught about disaster planning. Another study found that 69.75% of students understood what disaster preparedness entails [15]. The current findings reflect that students knew about disaster preparedness accidentally while only a few had received training or lectures from teaching staff about disaster preparedness. Additionally, it highlights the importance of involving teachers in educating and training students on how to deal with different types of disasters.

According to this study, fires were the most commonly identified disasters (89.7%), followed by earthquakes (77.8%). On contrary, the Indonesian study found that 90% were aware of earthquakes as the main type of disaster, with erosion and volcanic eruptions being the least known disasters [18]. This is most likely due to variations in the geographics of both countries. Indonesia is one of the countries that geologically has a meeting of three active tectonic plates which makes it prone to natural disasters [18].

In this study, social media and the internet were the most frequent sources of information reported by 78.8% and 69.7%, respectively. This was consistent with the results of another study that reported that internet media exerts a great influence on the source of disaster information [18]. Young people can easily use social media and the internet to get information.

The majority of the sample population (95.9%) had positive attitudes toward disaster management preparedness. Another study in Yemen showed lower results, demonstrating a moderate attitude score toward disaster management readiness [17]. This may be due to the differences in the sampled population and study settings.

The higher level of positive attitude among students in this study encourages the study investigators to recommend the necessity of establishing disaster preparedness and management committees in schools with the responsibility of educating and training all school members about disaster management, in addition to incorporating disaster preparedness in their curricula or elective training schedule.

Unfortunately, despite the positive attitude among students toward disaster management, there was an evident shortage in providing training on disaster preparedness and management as only 34.2% of the respondents were previously involved in a disaster drill(s) or workshop(s) in their school or city. This outcome is similar to that reported by El-Hosany and Ghonem in their Egyptian study. They found that 99.1% of the study group did not receive any disaster preparedness training previously despite an average level of awareness about this topic among the participants [19].

Furthermore, there was a significant association between good knowledge about disaster management preparedness and the female gender. This is inconsistent with another study that reported no significant association between gender and knowledge and attitude toward disaster management [19]. This finding is the same as an earlier study that showed that male students were more confused than female students about disaster management [20]. As female students are more interested to follow the news and browse social media and the internet, they possessed more knowledge in comparison to males.

Students with highly educated parents showed more knowledge scores. This outcome is logical as educated parents can correctly guide their children to be aware of different topics including disasters and their management. Moreover, students from high-income families were more knowledgeable than their peers, which may be due to their opportunity to possess smartphones and internet access, which, in turn, makes information more accessible. Finally, employed fathers showed a greater impact on their children’s level of knowledge, and this finding is consistent with that reported by Maleki et al. [21].

Respondents with high family incomes showed more positive attitudes than their peers. This could be due to the availability of kits needed for disaster management among high-income students. This category of students also possessed better knowledge scores which act as a guide for positive attitudes toward disaster preparedness.

This study had some limitations. First, the obtained data via an online approach may threaten the credibility of the data. Second, the cross-sectional design of the study limits determining causation. Third, students’ practice was better to be assessed to provide a full picture of their actual behavior on exposure to different disasters. Despite these limitations, this study had many strengths. Crucially, it highlighted the knowledge level of Saudi students about disaster preparedness as few previous studies had analyzed this critical issue. Second, this study was conducted on a large scale of secondary school students in many big cities such as Jeddah, Makkah, Al-Madinah, Al-Taif, and other cities in the western region. Third, this study is an initiative to guide other researchers to investigate more and collect in-depth data about this important topic through a quasi-experimental study in the form of active training (mock drill) for students and other school members to assess its impact on their practice. Finally, the present study attracts the attention of policymakers toward good preparation of the school community including students to face different disasters and the establishment of disaster management committees.

## Conclusions

Based on the analysis, it can be stated that the attitude and knowledge of high school students in the western region of Saudi Arabia were fair. However, disaster response training simulations for instructors and students must be included in the school curriculum. It is necessary to establish disaster preparedness and management committees inside the schools to educate and train all school members about disaster management. Intervention studies may enhance Saudi students’ disaster management readiness. Families also have a crucial responsibility to inform their children about disasters and their repercussions, as well as informing them about the actions to take in the lead-up to, during, and after a disaster. Future research should take into consideration the relevant factors discussed in this study.
